# Models of care for chronic myeloid leukemia patients during the COVID‐19 pandemic in the United Kingdom: Changes in patient attitudes to remote consultations and future implications

**DOI:** 10.1002/jha2.220

**Published:** 2021-05-14

**Authors:** Nicholas Duncan, Nigel Deekes, David FitzGerald, Tin Wai Terry Ng, Manoj Raghavan

**Affiliations:** ^1^ Queen Elizabeth Hospital and University of Birmingham Birmingham UK; ^2^ Patient representative; ^3^ School of Pharmacy University College London London UK

## Abstract

The ongoing COVID‐19 pandemic has presented numerous challenges to the provision of patient care within hematology. We undertook a questionnaire‐based study investigating the experiences and opinions of patients with chronic myeloid leukemia (CML) in the UK in relation to the different models of follow‐up care received during the pandemic. One hundred fifty‐four patients completed the online questionnaire. Only 19% of patients had experienced remote clinics prior to the pandemic compared with 91% afterward. After having experience of remote clinics, the proportion of patients who were positive about the remote clinic concept increased from 34% to 52% (P < .05). However, when asked to compare their experiences with face to face versus remote clinics, 48% preferred face‐to‐face clinics compared with 17% preferring remote clinics (35% expressed no preference). During the pandemic, frequency of blood tests was unchanged for 71% of patients, although they were performed in a number of different locations. The majority of patients (57%) had medication delivered to their home, with a small number (8%) having difficulty obtaining their medication. In terms of future models of care, 72% of patients were in favor of building remote appointments into the clinic model with 61% expressing a preference for a mixture of remote and face‐to‐face appointments. There was also interest in greater utilization of primary care for blood testing. Our findings should help optimize future models of care for CML patients.

## 1 INTRODUCTION

During the COVID‐19 pandemic, models of care for users of health services have had to change [1, 2, 3, 4], often at very short notice, and without the opportunity for in‐depth assessment of the pros and cons of such modifications in service provision [5]. There are limited published data on how the management of patients with chronic myeloid leukemia has changed during the pandemic [6], and it is not known how these changes in service delivery have impacted on the patient experience. From a UK perspective, follow‐up care for this patient population has historically been delivered along traditional lines, based around regular face‐to‐face outpatient hospital clinic appointments with a hematology specialist. At that appointment, patients would usually undergo both routine and disease‐specific blood tests and would also be prescribed and collect medication from the hospital pharmacy department. To the best of our knowledge, alternative models of care, particularly those utilizing remote clinic technology (eg, video or telephone clinics) were not well established for these patients, and limited previous research had indicated that (in principle at least), patients generally felt more comfortable with the face‐to‐face model that they were familiar with [7]. However, since the start of the COVID‐19 pandemic, one of the key drivers in the delivery of outpatient care across the health service has been to reduce footfall within the hospital environment and this is likely to have resulted in increases in the utilization of remote clinic models, not just in relation to appointments, but also in terms of patient monitoring and medication supply. This enforced change in service delivery, viewed in the context of the NHS Long‐Term Plan and its call for the delivery of more “person‐centered care” and for enhanced use of digital technologies such as telephone and video consultations, [8] provided an ideal opportunity to undertake a patient‐focused research study in this setting.

The aim of this questionnaire‐based study was to investigate the experiences and opinions of CML patients in the UK in relation to the different models of follow‐up care that they have been receiving during the COVID‐19 pandemic. We hypothesized that changes in service delivery would have had both positive and negative impacts on the patient experience and envisaged that our findings could be used by hematology centers within the UK to develop and refine future models of care that could be primarily driven by the user experience and not (as historically has been the case) by the needs of the service providers.

## 2 METHODS

To maximize the response rate in a time‐efficient manner, we decided to utilize a questionnaire‐based methodology for this study. An online questionnaire was prepared using the www.onlinesurveys.ac.uk survey tool. All study authors contributed to the design and content of the questionnaire. Two of the study authors were patients with CML: this ensured an appropriate level of patient and public involvement (PPI) in the research process. The questionnaire covered the following topics: patient demographics, clinic experience and logistics pre‐ and during COVID‐19, blood testing pre‐ and during COVID‐19, and medication supply pre‐ and during COVID‐19. The questionnaire contained 24 questions: the majority were closed questions with multiple choice answers, although respondents were given the opportunity to provide free text responses where appropriate. A news item with a link to the on‐line questionnaire was posted on two UK‐based CML patient support websites on August 1, 2020 and the link was kept open until August 31, 2020. Further promotion of the survey by the websites was undertaken 2 and 3 weeks after the survey opened to increase the number of responses.

Patients resident in the UK with a diagnosis of CML were eligible to complete the questionnaire. There were no additional exclusion criteria set. The first page of the on‐line questionnaire provided all relevant background information to the study (in lieu of a specific participant‐information sheet). From a consent perspective, before proceeding with the main body of the questionnaire, respondents were required to acknowledge electronically that they were happy to proceed and for their responses to be used in the study based on the information they had received. No patient identifiers were collected to ensure the anonymity of the questionnaire data. Ethical approval for the study was granted by the University of Birmingham Ethics Committee (ERN_20‐0982).

## 3 RESULTS

Completed questionnaires were received from 166 participants. Twelve participants were excluded from the analysis as they were not resident in the UK. Demographic data for the 154 remaining participants are presented in Table 1. The majority of respondents were female, over 90% were resident in England and there was an equal split between those diagnosed with CML within the past 5 years and those who were diagnosed more than 5 years ago.

**TABLE 1:** Participant demographics

	Number of respondents (%) (Total = 154)
**Gender**:	
Female	110 (71%)
Male	44 (29%)
**Age group**:	
16‐24	1 (1%)
25‐44	36 (23%)
45‐64	89 (58%)
65 and older	28 (18%)
**Country of residence**:	
England	140 (91%)
Scotland	7 (4%)
Wales	6 (4%)
Northern Ireland	1 (1%)
**Time since diagnosis**:	
<1 year	10 (6%)
1‐5 years	67 (44%)
6‐10 years	36 (23%)
>10 years	41 (27%)
**Current drug treatment**:	
Imatinib	58 (38%)
Dasatinib	38 (25%)
Nilotinib	34 (22%)
Bosutinib	14 (9%)
Asciminib	2 (1%)
Ponatinib	1 (1%)
Not on treatment	7 (4%)

Prior to being asked about how their clinic experience had changed since the start of the COVID‐19 pandemic, participants were asked a series of questions relating to their “usual” clinic experience. Key findings are summarized in Table 2.

**TABLE 2:** Clinic experience of CML patients prior to COVID‐19 pandemic

	Number of respondents (%)
**Health care professional(s) seen at clinic visits**:	
Doctor	137 (89%)
Nurse	43 (28%)
Pharmacist	16 (10%)
Other	4 (3%)
**Travel time to clinic**:	
<30 min	90 (58%)
30‐60 min	48 (31%)
>60 min	16 (10%)
**Time spent at hospital when attending clinic**:	
< 1 h	49 (32%)
1‐2 h	69 (45%)
2‐3 h	27 (18%)
>3 h	8 (5%)
**Frequency of clinic visits**:	
Monthly	11 (7%)
Every 2–3 months	121 (79%)
Every 4–6 months	17 (11%)
Other	4 (3%)

### 3.1 Clinic models before and during COVID‐19 pandemic

Prior to the COVID‐19 pandemic, 29 participants (19%) had experience of remote clinics for their CML management: in all cases, this had taken the form of a telephone consultation. Since the start of the pandemic, the number of participants who had experienced a remote clinic consultation increased to 140 (91%), Table 3. Frequency of clinic appointments had remained the same for 75% of respondents and 20% reported a reduction in frequency since the start of the pandemic.

**TABLE 3:** Breakdown of clinic appointments for CML patients during COVID‐19 pandemic

	Number of respondents (%)
**Type of clinic appointment experienced**:	
Face‐to‐face	23 (15%)
Telephone	136 (88%)
Video	4 (3%)
Not had a clinic appointment	8 (5%)

### 3.2 Opinions of different clinic models

Participants were asked to rank (on a scale of 0–10, where 0 = not at all interested and 10 = extremely interested) their interest in having remote clinic appointments as an alternative to face to face appointments prior to the COVID‐19 pandemic. One hundred fifty‐two participants responded and the mean score was 4.4, 95% CI [3.80, 5.01]. Fifty‐two participants (34%) recorded a score of 6–10 indicating a positive opinion of the remote clinic concept. Thirty percent of respondents stated that they were not at all interested in remote clinic appointments prior to the pandemic.

Participants who had experienced a remote clinic consultation during the COVID‐19 pandemic were then asked to rank (using the same scale) their interest in continuing to have remote clinic appointments in the future. One hundred thirty‐six participants answered this question and the mean score was 5.8, 95% CI [5.21, 6.41]. Seventy‐one participants (52%) recorded a score of 6–10 indicating a positive opinion of the remote clinic concept. For those participants who answered both questions (n = 136), the mean score increased from 4.4 to 5.8 (*P* < .00001, 95% CI for the difference [0.89, 1.85], paired t‐test)

Patients who had been diagnosed >5 years previously were significantly more likely to be interested in remote clinic appointments at baseline than those with a more recent diagnosis of CML (mean score 5.1 vs 3.8 *P* = .038, 95% CI for the difference [0.07, 2.58] unpaired *t*‐test). They were also more likely to be interested in continuing to have remote clinic appointments in the future (mean score 6.3 vs 5.3), although the difference was not statistically significant (*P* = .13, 95% CI for the difference [‐0.28, 2.13], unpaired *t*‐test).

When participants were asked to compare their experiences with face to face and remote clinic models, 48% stated that they preferred the face to face model, 17% preferred the remote clinic model, and 35% had no preference. The most commonly expressed reasons for preferring one model to another are outlined in Table 4.

**TABLE 4:** Reasons for clinic model preference expressed by CML patients

	Number of respondents (%)
**Reasons for preferring face to face clinic (n = 71)**	
Easier to discuss concerns in person	56 (79%)
Reassuring to see my CML specialist in person	46 (65%)
It is a simpler model	37 (52%)
Like to be able to talk to other patients and/or HCPs while I am at the hospital	14 (20%)
Practical difficulties with remote clinic technology	8 (11%)
**Reasons for preferring remote clinic (n = 25)**	
More convenient because I did not need to travel	24 (96%)
Felt safer not coming to the hospital	18 (72%)
Financially better	14 (56%)
More convenient because I did not need to arrange time off work	7 (28%)
More convenient because I did not need to arrange childcare	5 (20%)

Finally, participants were asked their opinions of various clinic models that could be followed in the future. The most popular option, favored by 61% of respondents, was a mixture of face‐to‐face and remote appointments (Table 5).

**TABLE 5:** CML patient preferences for future clinic models

	Number of respondents (%)
Prefer all appointments to be remote	17 (11%)
Prefer all appointments to be face to face	42 (28%)
Prefer a mixture of the above:	94 (61%)
Equal split	50
Mainly remote	35
Mainly face to face	8

### 3.3 Blood tests

Participants were asked about their experience of blood test monitoring before and during the pandemic. Responses in relation to location of blood tests are summarized in Table 6. It can be seen that there was an increase in utilization of GP surgeries and “other” locations for blood testing since the start of the pandemic. Examples under the “other” category included: walk in center, home testing, and pop‐up testing center.

**TABLE 6:** Location and timing of bloods tests for CML patients

	Number of respondents (%)	
	Before (n = 153)	During (n = 154)
At the hospital where I attend clinic – on the day of clinic	81 (53%)	31 (20%)
At the hospital where I attend clinic – set time in advance	42 (28%)	55 (36%)
At a hospital closer to home – set time in advance	7 (5%)	9 (6%)
At my GP surgery – set time in advance	11 (7%)	28 (19%)
Other	12 (8%)	25 (16%)
N/A – not had blood tests	0	6 (4%)

When asked about frequency of blood tests since the start of the pandemic, the majority of respondents (71%) stated that the frequency of testing had not changed. Nine percent stated that the frequency had increased, 18% that it had decreased, and 2% were unsure. Six patients stated that they had not received any blood tests. Of those patients (n = 28) who stated that the frequency had decreased, the majority (64%) stated that both their routine blood tests and their BCR‐ABL PCR tests had been affected, with a further 18% stating that just their BCR‐ABL PCR testing frequency had reduced.

Twenty‐four participants (16%) stated that they had experienced problems with blood tests during the COVID‐19 pandemic. The majority of problems related to difficulties getting appointments for blood tests, alternative providers not being familiar with the specialist blood tests required for CML patients and blood samples not being processed.

Participants were also asked to express a preference for different blood testing models in the setting of remote clinic appointments. Table 7 summarizes the responses.

**TABLE 7:** CML patient preferences for location and timing of blood tests in the future

	Number of respondents (%)
At the hospital where I attend clinic – set time in advance	59 (39%)
At a hospital closer to home – set time in advance	21 (14%)
At my GP surgery – set time in advance	45 (29%)
Other	8 (5%)
No preference	10 (6%)
N/A – I would not want remote appointments	10 (6%)

### 3.4 Medication supply

Prior to the COVID‐19 pandemic, the majority of patients (68%) who were receiving medication for their CML collected their prescription from the hospital where they attended the CML clinic. Twenty‐five percent of patients had their medication delivered to their home. The remainder collected it from a different hospital or from their community pharmacy.

Since the pandemic, the percentage of patients receiving home delivery of their medication increased to 57% with only 34% collecting it from the hospital where they attended clinic. There was no change in the percentage of patients collecting medication from a different hospital or from a community pharmacy. Twelve participants (8.3%) stated that they had experienced problems with obtaining their CML medication during the COVID‐19 pandemic. The majority of problems related to drug unavailability.

## 4 DISCUSSION

To our knowledge, this is the first study to investigate the impact of the COVID‐19 pandemic on provision of care for patients with CML from a patient perspective. From a demographic point of view, the make‐up of the 154 respondents was seen to be fairly representative of the UK CML population, although there was a higher than expected bias in favor of female patients, given that the UK incidence is slightly higher in males (1.5 per 100 000 versus 1.2 per 100 000) [9]. For the majority of patients, clinic experiences prior to the COVID pandemic followed the traditional model of a face‐to‐face consultation with a specialist doctor every 2–3 months. Given this study's focus on remote clinic models, it was noteworthy that >10% of respondents had to travel for more than 1 h to get to clinic and nearly a quarter of patients stated that they spent on average >2 h at the hospital when they attended for clinic appointments.

Despite increasing interest in technology‐based patient consultations [10] in cancer care prior to the COVID pandemic [11, 12], uptake of such models in UK CML practice appears to have been relatively low with only 19% of respondents reporting experience with telephone consultations and no patients having experienced a video consultation. Interestingly, only a third expressed a positive baseline opinion in terms of their level of interest in having remote clinic appointments. As recommended elsewhere [1, 2, 3, 4], a marked increase in the utilization of remote clinic technology was reported since the start of the pandemic, with 91% of respondents having subsequently experienced at least one telephone or video consultation. Importantly and reassuringly, we demonstrated that the increased exposure to remote appointments led to a statistically significant increase in participant's level of interest in this model of care with a majority now expressing a positive opinion in relation to having remote appointments in the future. Patients who had been diagnosed >5 years ago were more positive about the remote clinic model than those with a more recent diagnosis. This was to be expected as such patients are more likely to be stabilized on a specific drug, and to have achieved a deep molecular response and therefore may be less in need of the additional reassurances potentially offered by a face to face consultation. The generally favorable findings in relation to opinions of remote consultations match those published elsewhere [13, 14].

When asked to compare experiences of face‐to‐face versus remote clinics, almost 50% preferred the former with only 17% preferring the remote model. Respondents rated the ability to discuss concerns in person as the number one reason for preferring the face‐to‐face model. Although only 25 respondents expressed a preference for the remote model, the convenience factor associated with not having to travel to the hospital was the most commonly expressed reason in favor of this approach. However, there was no correlation between travel time to clinic and clinic preferences. In terms of future models of care, over 60% of respondents expressed a preference for a mixture of face‐to‐face and remote appointments with a 50:50 or 75:25 (in favor of remote) split being the most popular options, indicating that a one size fits all approach is unlikely to be the best way forward in relation to clinic configuration.

It was reassuring that for the survey population as a whole, only 18% reported a reduction in frequency of blood testing since the start of the pandemic with less than one in six patients stating that their molecular monitoring had been negatively affected. However, it is concerning that 4% of respondents had not had any blood tests undertaken. Both before and during the pandemic, there was a degree of variation in terms of both location and timing of blood tests although the majority of respondents were still having blood tests performed at the hospital where their CML clinic was based. When asked about preferences for future provision of blood tests in the setting of remote clinic appointments, it was striking that nearly one‐third of patients expressed a preference for their blood tests to be undertaken at their GP surgery. This was a marked increase on the 7% of patients who were actually having blood tests at the GP surgery prior to the pandemic. However, for those centers looking to develop this remote model, the survey responses indicate that there may be a number of staff training and logistical challenges to be overcome before being in a position to confidently move testing away from the CML center.

In line with previously published recommendations and experiences reported elsewhere [2, 15], the use of home delivery of medication more than doubled in the survey population during the pandemic, with relatively few reported problems with the medication supply process. Those problems that did arise tended to relate to local stock availability issues. On‐going delivery of medication from hospital pharmacy departments, although advantageous on a number of fronts, does however present both economic and workload challenges for individual hospitals. It was noticeable that both before and during the pandemic, few patients collected their medication from a community pharmacy and consideration could be given in the future to exploring increased utilization of community pharmacies [16] as a means of supplying medication to CML patients.

A limitation of the study was the online methodology that may have resulted in a bias in favor of more technologically literate patients. It was also not possible to achieve the comparable depth of responses as would have been achieved through an interview or focus‐group based methodology and this is something that could be explored in future work. Furthermore, the time period of our study was relatively short (approximately 5 months) so data on changes to frequency of clinic appointments and blood tests should be interpreted with caution.

In conclusion, patients with CML are in the majority positive regarding the move to remote monitoring but there is a preference for a mixed economy of remote and face‐to‐face consultations that could be implemented when the pandemic has subsided. Increased utilization of primary care and community pharmacy models for blood testing and medication supply should also be considered.

## CONFLICT OF INTEREST

The authors have no conflict of interest.

## AUTHOR CONTRIBUTIONS

NDu, NDe, DF, TWTN, and MR designed the research study. NDu, NDe, and DF performed the research. NDu analyzed the data and wrote the paper. All authors were involved in revisions and approved the final draft.

## Data Availability

The data that support the findings of this study are openly available [17] in Figshare at: https://doi.org/10.6084/m9.figshare.14074499.v1
